# 1739. Mumps Vaccine Hesitancy: Current Evidence and An Evidence-Based Campaign in Japan

**DOI:** 10.1093/ofid/ofad500.1570

**Published:** 2023-11-27

**Authors:** Satoko Ugai, Tomotaka Ugai, Tetsuya Kanayama, Hajime Kamiya, Akihiko Saitoh, Natalie Slopen

**Affiliations:** Harvard T.H. Chan School of Public Health, Brookline, Massachusetts; Brigham and Women's Hospital, Brookline, Massachusetts; Tokamachi Hospital, Tokamachi, Niigata, Japan; Infectious Diseases Surveillance Center, National Institute of Infectious Diseases, Sinjyuku, Tokyo, Japan; Niigata University, Niigata, Niigata, Japan; Harvard T.H. Chan School of Public Health, Brookline, Massachusetts

## Abstract

**Background:**

Mumps is still endemic in Japan because mumps vaccination is voluntary. In this study, we investigated associations of parental socioeconomic status, family structure, and knowledge/belief about mumps and mumps vaccine with parental decision to vaccinate their children using a questionnaire survey; and, we evaluated effectiveness of a campaign based on the survey results.

**Methods:**

We conducted a cross-sectional survey of parents with children aged 1 to 6 years attending preschools or kindergartens in Tokamachi City, Japan. We assessed the association of parental factors with their decision to vaccinate their children using multivariable logistic regression analyses. We designed a campaign, based on the survey results and compared the number of provided mumps vaccinations before and after the campaign using a trend test.

**Results:**

In total, 1391 of 1617 (86%) eligible parents completed the survey. Among these parents, 229 (16%) vaccinated their children. In multivariable analyses, higher parental education (odds ratio (OR) = 2.21; 95% CI, 1.59-3.08; P < 0.001), greater knowledge about mumps and the mumps vaccine (OR=1.88; 95% CI, 1.60-2.21; P < 0.001), and living without grandparents (OR=1.44; 95% CI, 1.05-1.99; P= 0.024) were significantly associated with parental decision to vaccinate their children. The number of provided mumps vaccinations increased dramatically following the campaign (P < 0.001).

**Conclusion:**

Clear explanations of mumps and mumps vaccines from pediatricians may be an effective strategy to increase vaccination coverage. Further research is needed to examine the relevance of our findings to the uptake of other voluntary vaccines among children, as well.

**Disclosures:**

**All Authors**: No reported disclosures

